# Dual Challenges: Managing Schizophrenia Relapse in a Patient with Sickle Cell Anemia

**DOI:** 10.1192/j.eurpsy.2025.1209

**Published:** 2025-08-26

**Authors:** S. Ben Aissa, C. Najar, K. Razki, M. Cheour, F. Fekih-Romdhane

**Affiliations:** 1 Razi Hospital, LaManouba; 2 Faculty Of Medicine of Tunis, Tunis El Manar University, Tunis, Tunisia

## Abstract

**Introduction:**

Sickle cell anemia (SCA) is a genetic disorder resulting in chronic hemolysis and vaso-occlusive crises, which can lead to severe systemic complications. Schizophrenia is a major psychiatric disorder characterized by persistent psychotic symptoms, including delusions and hallucinations. The intersection of these two conditions presents unique clinical challenges, as the management of SCA may complicate the treatment of schizophrenia and vice versa. Despite the prevalence of such comorbid cases, there is a significant gap in research addressing the complexities of managing these conditions concurrently.

**Objectives:**

This case report aims to describe a rare and complex case of schizophrenia in a patient with sickle cell anemia, including the clinical presentation, diagnosis, and management. Additionally, it seeks to highlight the challenges of managing these co-occurring conditions and to promote further research into integrated treatment strategies for such comorbidities.

**Methods:**

We present a 32-year-old male with a history of sickle cell anemia and schizophrenia who was admitted to the psychiatric department at Razi Hospital, LaManouba. The patient was admitted following a suicide attempt driven by acute psychotic symptoms, including delusions and auditory hallucinations commanding self-harm. Upon admission, he was started on a regimen of benzodiazepines to manage severe anxiety and antipsychotic medication to address the hallucinations and delusions. The initial physical examination revealed no significant abnormalities. On the third day of hospitalization, the patient began exhibiting respiratory symptoms, including chest pain and difficulty breathing, which prompted further diagnostic evaluation.

**Results:**

On the third day of hospitalization, the patient developed respiratory symptoms including chest pain, fever, and dyspnea. Evaluation, including lab tests and imaging, revealed acute chest syndrome. Given the risk of benzodiazepines worsening respiratory issues, their use was discontinued, and Haloperidol was introduced at 5 mg to manage psychotic symptoms, with close monitoring for suicidal ideation. The hematology and pneumology departments initiated a treatment protocol involving oxygen therapy, intravenous hydration, paracetamol for pain management, and antibiotics (ceftriaxone and clarithromycin). Within a week, both psychiatric and respiratory symptoms significantly improved, leading to a reduction in supplemental oxygen and discharge with a comprehensive follow-up plan.

**Conclusions:**

This case underscores the complexity of managing co-occurring schizophrenia and sickle cell anemia, highlighting the need for a multidisciplinary approach. Integrated treatment strategies are crucial for effectively addressing both psychiatric and medical complications in such comorbid conditions

**Disclosure of Interest:**

None Declared

